# The Interplay Between Exosomes and Gut Microbiota in Neuroinflammation: A New Frontier in Alzheimer’s Disease

**DOI:** 10.3390/ijms26125828

**Published:** 2025-06-18

**Authors:** Sara Uceda, Manuel Reiriz, Víctor Echeverry-Alzate, Ana Isabel Beltrán-Velasco

**Affiliations:** NBC Group, School of Life and Nature Sciences, Nebrija University, 28240 Madrid, Spain; suceda@nebrija.es (S.U.); mreiriz@nebrija.es (M.R.); vecheverry@nebrija.es (V.E.-A.)

**Keywords:** exosomes, Alzheimer’s disease, gut microbiota, neurodegeneration, biomarkers, therapy, diagnostics, nanotechnology

## Abstract

Alzheimer’s disease (AD) is a complex neurodegenerative condition that is characterized by the accumulation of amyloid-β, the hyperphosphorylation of tau, and persistent neuroinflammation. However, these hallmarks alone do not fully capture the intricacies of AD pathology, thus necessitating the investigation of emerging mechanisms and innovative tools. Exosomes (nanoscale vesicles involved in cell communication and immune modulation) have emerged as pivotal cellular vehicles due to their dual role—both in the propagation of pathological proteins and the regulation of inflammatory responses. Furthermore, these vesicles have been demonstrated to play a crucial role in the mediation of the effects of microbiota-derived metabolites and the reflection of systemic influences such as dysbiosis, thereby establishing a link between the gut–brain axis and the progression of AD. A comprehensive narrative literature review was conducted using the following databases: ScienceDirect, Scopus, Wiley, Web of Science, Medline, and PubMed, covering studies published between 2015 and 2025. Inclusion and exclusion criteria were established to select research addressing exosomal biogenesis, their functional and diagnosis role, their therapeutic potential, and the emerging evidence on microbiota–exosome interplay in Alzheimer’s disease. Exosomes have been identified as integral mediators of intercellular communication, reflecting the molecular state of the central nervous system. These particles have been shown to promote the propagation of pathological proteins, modulate neuroinflammatory responses, and serve as non-invasive biomarkers due to their detectability in peripheral fluids. Advances in exosomal engineering and microbiome-based interventions underscore the potential for targeting systemic and CNS-specific mechanisms to develop integrative therapies for AD. Exosomes present a promising approach for the early diagnosis and personalized treatment of Alzheimer’s disease. However, methodological challenges and ongoing controversies, including those related to the influence of systemic factors such as dysbiosis, necessitate multidisciplinary research to optimize and standardize these strategies.

## 1. Introduction

Alzheimer’s disease is the most prevalent form of dementia and is considered one of the most significant public health challenges worldwide. The aging population has led to a notable increase in the incidence of this neurodegenerative disease, placing considerable strain on healthcare systems and underscoring the pressing need for a comprehensive understanding of its pathological mechanisms [1,2]. Conventional knowledge attributes the pathogenesis of AD to the accumulation of amyloid plaques derived from amyloid-β (Aβ) and the formation of neurofibrillary tangles composed of hyperphosphorylated tau protein [3]. These processes trigger synaptic loss and neuronal dysfunction, contribute to chronic activation of the inflammatory response in the central nervous system (CNS), and ultimately lead to neuronal death [4]. Despite the considerable time and resources that have been devoted to research over several decades and the development of therapeutic interventions that target these two proteins, clinical outcomes have been limited, underscoring the necessity to explore alternative approaches that can intervene earlier and more specifically in the onset and progression of the disease [5].

Recent findings have contributed to an augmentation of our comprehension of AD, leading to a divergence from the prevailing amyloid–tau paradigm. AD is now recognized as a multifactorial and heterogeneous disorder, with distinct pathological subtypes (ranging from amyloidogenic forms to variants dominated by tau pathology or altered brain network connectivity) that underscore the complex interplay between protein accumulation, metabolic dysfunction, and neuroinflammation [6,7]. Moreover, metabolic, immune, and vascular factors are key contributors in the early stages of the disease. Cerebral hypometabolism, as revealed by positron emission tomography (PET) studies, and blood–brain barrier (BBB) disruptions further contribute to neuronal stress and irreversible cellular damage [8]. The interplay of these multisystem alterations is further modulated by factors such as cognitive reserve, which can delay the onset of clinical symptoms despite significant neuropathology.

A novel aspect of AD pathophysiology is the emerging role of the intestinal microbiota, where dysbiosis—characterized by an increase in pro-inflammatory bacteria and a decrease in anti-inflammatory, short-chain fatty acid (SCFA)-producing bacteria—has been observed [9]. This microbial imbalance leads to an altered metabolite profile, including increased levels of lipopolysaccharides (LPSs), trimethylamine-N-oxide, and pro-inflammatory cytokines, alongside a reduction in beneficial metabolites such as butyrate [10,11]. These changes can induce a systemic pro-inflammatory state via the gut–brain axis, directly impacting brain function and modulating both the biogenesis and molecular cargo of exosomes. In this regard, bacterial metabolites, particularly SCFAs, exhibit neuroprotective and anti-inflammatory properties, suggesting that gut-derived signals and exosomal communication play a crucial role in AD progression. Furthermore, environmental factors and genetic predispositions influence microbiota composition, potentially contributing to early alterations in gut–brain communication that preceded the onset of overt cognitive decline in AD [12].

Recent research has identified extracellular vesicles, particularly those termed exosomes, as pivotal mediators of intercellular communication within the CNS [13,14]. Exosomes are nanovesicles measuring between 30 and 150 nanometers in diameter, released by virtually all cell types, and their composition reflects that of their cell of origin [15]. These vesicles have been shown to contain proteins, lipids, microRNAs, and other bioactive molecules [16]. While initially regarded as mere cellular debris, exosome research has advanced to a point where they are now recognized as complex signaling entities capable of regulating cellular functions remotely and modulating immune and neuroinflammatory responses [17,18].

A molecular perspective reveals the dual role of exosomes in AD. Exosomes act as a conduit for the distribution of pathological agents, including fragments of the amyloid β peptide and oligomeric or aggregated forms of tau [19]. This process facilitates the spread of these substances between neurons, thereby amplifying neurodegenerative damage [20]. Several in vitro and in vivo studies have shown that the internalization of exosomes loaded with the above pathological agents can induce degenerative responses in recipient cells, suggesting a seeding or initiation mechanism that could elucidate the characteristic progression of the disease through specific neuronal circuits [21,22]. Conversely, it has been proposed that exosomes could also be involved in neuroprotective mechanisms and in the regulation of the immune response, depending on the context and the molecular load they carry [23,24]. This complexity is challenging traditional therapeutic strategies, allowing for more precise, individualized interventions.

The capacity of exosome circulation in biological fluids such as plasma and cerebrospinal fluid has generated significant interest in their potential as minimally invasive biomarkers [25]. The detection and analysis of exosome biomarkers in patient samples offer the possibility of identifying molecular signatures associated with AD at very early stages, long before the onset of obvious clinical symptoms [26]. The development and refinement of isolation and characterization techniques, encompassing ultracentrifugation, immunoaffinity methods, nanotechnology-based approaches, and high-resolution flow cytometry, have enabled a more precise delineation of their protein and genetic profiles [27,28]. This methodological progression facilitates the identification of specific biomarkers and enables the exploration of biogenesis, cargo targeting, and secretion mechanisms of exosomes under pathological conditions [29].

The present narrative review examines selected molecular and cellular mechanisms of exosome biogenesis and release. These mechanisms include pathways—such as microbiota-derived signals and short-chain fatty acids—that have been implicated in exosomal cargo sorting and in the modulation of neuroinflammatory processes in Alzheimer’s disease. Also, the experimental evidence linking the presence of Aβ- and tau-loaded exosomes with the progression of neurodegeneration in AD is discussed, with particular emphasis on studies correlating these findings with alterations in synaptic function and the activation of inflammatory processes in the CNS.

In addition, the present study explores the potential of exosome analysis as a diagnostic tool, highlighting the advantages and challenges associated with their use as biomarkers. Finally, the study discusses emerging strategies that exploit the therapeutic potential of these vesicles for targeted delivery of drugs or modulatory molecules. These strategies also address the controversies and gaps in the interpretation of their dual roles in AD.

## 2. The Intestinal Microbiota and Its Metabolites in the Pathophysiology of Alzheimer’s Disease

The intestinal microbiota represents a complex and dynamic ecosystem comprising trillions of microorganisms inhabiting the gastrointestinal tract. These microorganisms encompass bacteria, viruses, fungi, and archaea. Under healthy conditions, the microbiota plays essential roles in maintaining homeostasis within the organism. It assists in nutrient digestion, vitamin production, and immune system regulation. Moreover, it serves as a critical modulator of the gut–brain axis, a bidirectional communication system involving endocrine, immune, and neural pathways. The microbiota has the capacity to influence brain function through the production of bioactive metabolites, which affect neurogenesis, synaptic plasticity, and neuroinflammation [12,30].

However, the composition of the intestinal microbiota is not static and can be altered by factors such as diet, aging, antibiotic use, and metabolic diseases, among others. These alterations can lead to a state of dysbiosis, characterized by a reduction in beneficial bacteria and an increase in pathogenic species [31]. In the context of AD, recent studies have shown that intestinal dysbiosis can trigger a systemic proinflammatory environment that affects the homeostasis of the CNS, contributing to neurodegeneration [32,33,34]. A decrease in SCFA-producing bacteria has been identified in patients with AD, as well as an increase in proinflammatory species that favor microglia activation and oxidative stress in the brain [35].

It is well established that microbiota-derived metabolites play an essential role in the regulation of multiple physiological and pathological processes. Among them, SCFAs (mainly acetate, propionate, and butyrate) are recognized for their anti-inflammatory and neuroprotective properties [36]. These compounds are produced through the fermentation of non-digestible dietary fibers by commensal bacteria, such as *Faecalibacterium prausnitzii* and *Bacteroides* spp. SCFAs have been shown to have a positive effect on the intestinal barrier, strengthening the junction between enterocytes and reducing the translocation of bacterial endotoxins into the bloodstream [37].

At the CNS level, SCFAs have been demonstrated to modulate microglia activation, reducing the production of proinflammatory cytokines such as interleukin-6 (IL-6) and tumor necrosis factor-alpha (TNF-α) [38]. Additionally, these metabolites can influence the synthesis of neurotransmitters, such as serotonin and GABA, by regulating the expression of genes related to their metabolism [39]. Other bioactive compounds derived from the microbiome, such as tryptophan metabolites like quinurenine and indoxylsulfate, have been found to cross the BBB and impact neuronal function [40]. Alterations in the levels of these metabolites have been associated with synaptic dysfunction, oxidative stress, and neuroinflammation in experimental models of AD [41]. Recent research has indicated that these metabolites also modulate exosome biogenesis and molecular cargo. SCFAs have been observed to influence the loading of bioactive molecules into exosomes, favoring the inclusion of anti-inflammatory and neuroprotective factors [42,43].

Dysbiosis impairs intestinal barrier function, resulting in the influx of bacterial byproducts, including LPS (an endotoxin produced by Gram-negative strains), into the bloodstream. LPS serves as a significant activator of the innate immune system, inducing macrophage activation and elevated levels of inflammatory cytokines. Increased circulating levels of LPS have been identified in patients with AD, suggesting that intestinal dysbiosis may act as a trigger for neuroinflammation [44].

A dysbiotic gut microbiota has been linked to the enrichment of exosomes with pro-inflammatory microRNAs and proteins that exacerbate neuroinflammation. Notably, exosomes originating from the gut microbiota of AD patients have been shown to induce tau hyperphosphorylation and aggregation in vitro, pointing to a possible mechanism for disease progression. These microbiota-derived exosomes may also contribute to cognitive decline in AD by promoting neuroinflammation and compromising the integrity of the BBB [45,46].

Furthermore, the activation of the gut–brain axis in a dysbiosis context has the potential to compromise the function of the BBB. This compromise may facilitate immune cell infiltration and the amplification of inflammation in the CNS [47]. Previous studies have demonstrated that the administration of SCFAs can restore the integrity of the BBB and reduce the accumulation of Aβ [48,49]. These findings suggest a protective role for these metabolites.

Intestinal dysbiosis has also been demonstrated to influence the production of neurotransmitters and neurohormones, thereby impacting cognitive function and emotional state [50]. Altered serotonin and GABA synthesis have been posited as a potential contributor to the onset of neuropsychiatric symptoms associated with AD, including anxiety and depression [51,52]. Collectively, these findings underscore the notion that the gut microbiome and its metabolites exert a significant influence on the pathophysiology of AD and represent a promising therapeutic target for the formulation of preventive and therapeutic strategies for this disease.

## 3. Exosomes in the Central Nervous System (CNS) and Their Connection with the Gut Microbiota

**Figure 1:**
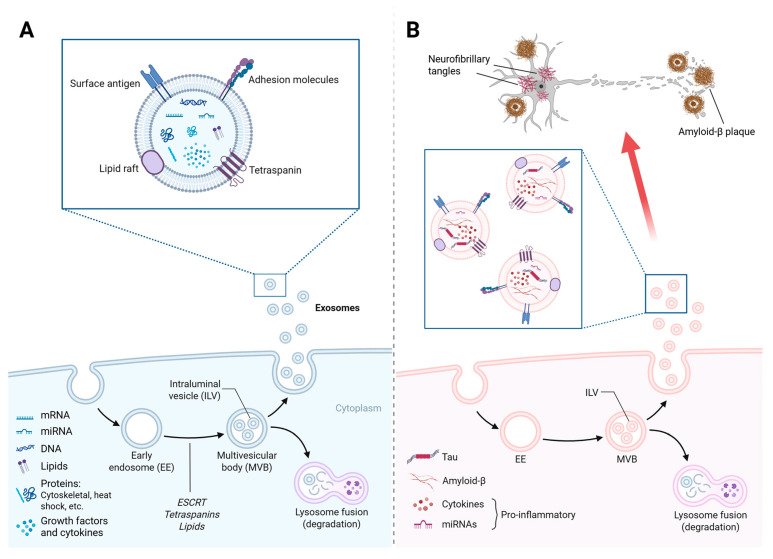
Exosome biogenesis and composition in healthy (**A**) and Alzheimer’s disease (**B**) conditions. The red arrow in (**B**) represents the spread of exosomes containing Tau and Aβ to other neurons. Tau and Aβ proteins aggregate to form neurofibrillary tangles and amyloid-β plaques, respectively. mRNA: messenger RNA, miRNA: microRNA, DNA: deoxyribonucleic acid, Tau: Tau protein, and Amyloid-β: amyloid beta protein. Created with BioRender.com.

Exosomes are a subset of extracellular vesicles with diameters ranging from 30 to 150 nanometers that play an essential role in intercellular communication within the CNS. Their biogenesis occurs in the endosomal compartment, through the formation of multivesicular bodies (MVBs), which, upon fusion with the plasma membrane, release their contents in the form of exosomes into the extracellular space [53]. This process is subject to rigorous regulation by the endosomal sorting complex required for transport (ESCRT) and alternative ceramide- and tetraspanin-dependent mechanisms, such as CD63 and CD81 (Figure 1A). The release of these vesicles is a dynamic process that responds to various cellular and environmental stimuli, including intense neuronal activity, hypoxia, and oxidative stress [54,55]. These stimuli influence the amount and molecular composition of the secreted exosomes. Research in this line has revealed that neuronal activity stimulates their production, thereby facilitating synaptic communication and modulating homeostatic processes. In contrast, pathological conditions, such as cell damage, induce changes in exosomal load, which, depending on the context, can promote inflammatory or neuroprotective responses [56]. For example, patients with inflammatory bowel disease present exosomes enriched with pro-inflammatory compounds such as IL-6, IL-8, and TNF-α [57] compared to those of healthy controls. Under normal conditions, however, exosomes contribute to the maintenance of gut homeostasis by modulating resident immune cells, the gut microbiota, and barrier integrity [58].

In neurodegenerative diseases such as AD, exosome levels are indicative of the pathological state of the cells of origin and act as mediators in the progression of the disease. It has been demonstrated that exosome levels can facilitate the propagation of pathological proteins, such as beta-amyloid fragments and abnormal forms of tau, contributing to progressive neuronal deterioration [26,59]. Furthermore, they have been implicated in the regulation of neuroinflammation by transporting IL-6 and TNF-α, which foster a proinflammatory and neurotoxic environment, amplifying cell damage (Figure 1B). However, the significance of these vesicles in AD pathology appears to be contingent on the nature of the exosomal content and the physiological state of the organism [60]. This suggests the potential for external factors, such as the intestinal microbiota, to influence the composition and function of these exosomes in the CNS.

In this line, it has recently been observed that the gut microbiota can modulate exosomal biogenesis in the brain, thereby establishing a connection between the gut–brain axis and exosome-mediated intercellular communication [61]. Specifically, microbiome-derived metabolites, such as SCFAs, have been demonstrated to play a crucial role in regulating exosomal content [42,62]. A balanced gut microbiome has been shown to favor the production of anti-inflammatory and neuroprotective exosome profiles, while dysbiosis has been demonstrated to alter their content, increasing the presence of proinflammatory microRNAs and proteins that exacerbate neuronal damage [63,64,65]. SCFAs have been observed to modulate the expression of genes involved in exosomal loading and microglia activation, influencing the progression of brain inflammation and the CNS immune response [66].

In addition to regulating exosomal cargo, SCFAs have been shown to influence the membrane stability of vesicles, modifying their lipid composition and facilitating their interaction with target cells in the brain. It has been proposed that these metabolites may affect the internalization of vesicles by neurons and glial cells, modulating the propagation of inflammatory and neuroprotective signals in the CNS [67,68]. In a similar manner, other microbiome-derived compounds, such as tryptophan metabolites and polyamines, have been demonstrated to regulate exosomal cargo, thereby promoting biochemical profiles associated with either neuroprotection or inflammation, contingent upon the state of the microbiome [69].

Recent studies have demonstrated that alterations in the gut microbiota are associated with changes in the exosomal composition of the CNS. An increased presence of exosome-enriched inflammatory proteins and beta-amyloid fragments has been detected under conditions of dysbiosis, suggesting that the gut microbiome contributes to Alzheimer’s progression through the modulation of exosomal communication [42,70,71]. Elucidation of the mechanisms by which the microbiota regulates exosomal biogenesis and its influence on neuroinflammation will facilitate the development of more-precise therapeutic strategies.

The characterization of exosomal content in biological fluids has the potential to serve as an early biomarker for the disease, facilitating the identification of alterations prior to the clinical manifestation of the disease [72]. Moreover, the manipulation of the microbiome and exosomal engineering could constitute complementary approaches in the pursuit of targeted and personalized therapies against AD. 

## 4. Exosome-Mediated Regulation of Neuroinflammation and Gut Dysbiosis in AD

Neuroinflammation is marked by the dysregulated activation of microglia and astrocytes, leading to a persistent inflammatory state that exacerbates neuronal dysfunction and synaptic degeneration [73]. Through the transportation of proinflammatory cytokines and regulatory microRNAs, exosomes can amplify microglial activation, intensifying the production of inflammatory mediators and TNF-α. This intercellular communication further exacerbates the neuroimmune response, thereby fostering an environment that perpetuates synaptic and neuronal dysfunction [74,75].

A salient mechanism in this process is the interaction between immune-derived exosomes (i.e., exosomes of peripheral origin) and those of the CNS [76]. It has been identified that exosomes derived from immune cells and the intestinal epithelium under conditions of dysbiosis can traverse the BBB and induce a neuroinflammatory response in the CNS. These exosomes, containing LPS and other microbial components, have been shown to activate Toll-like receptors (TLRs) on microglia, thereby inducing a state of sustained inflammation [77,78].

Beyond the activation of TLRs, exosomes enriched with proinflammatory cytokines and damage-associated molecular patterns (DAMPs) have been demonstrated to influence downstream signaling cascades such as the MAPK and NF-κB pathways [79]. These cascades exacerbate microglial activation, promoting the release of reactive oxygen species (ROS) and further amplifying neuroinflammatory damage [80]. Additionally, the role of exosomal lipid rafts in facilitating intercellular communication has gained attention. These lipid domains enhance the binding and uptake of vesicles by recipient cells, ensuring the efficient transmission of inflammatory signals in AD [81]. Conversely, exosome production under anti-inflammatory conditions has been observed to carry neuroprotective factors such as IL-10 and transforming growth factor-beta (TGF-β), which counteract inflammation and facilitate tissue repair, thereby demonstrating their dual regulatory capacity [82,83].

**Figure 2:**
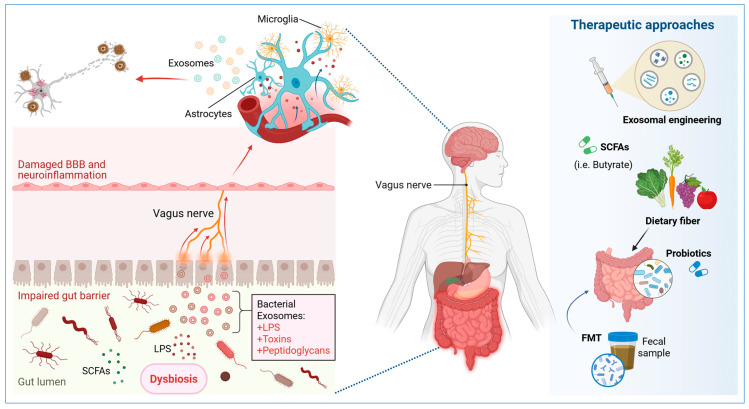
Exosomes and the gut–brain axis relationship in Alzheimer’s disease and therapeutic approaches including strategies that modulate the intestinal microbiome and exosome-based interventions. BBB: blood–brain barrier, FMT: fecal microbiota transplantation, LPS: lipopolysaccharides, SCFAs: short-chain fatty acids. Created with BioRender.com.

Emerging evidence indicates that gut-microbiota-derived metabolites, particularly SCFAs, modulate the biophysical properties of exosomes, affecting their stability and interactions with CNS cells [84]. Dysbiosis-associated alterations in SCFA profiles have been linked to modified exosomal content, particularly an increase in pro-inflammatory microRNAs and proteins, with consequent impacts on neuroinflammatory pathways (Figure 2). Particularly, the presence of butyrate has been demonstrated to induce alterations in the composition of exosomal membrane lipids, specifically with respect to the ratio of phospholipid to cholesterol. The effects of lipids on exosome dynamics are dependent on the metabolic environment, which can result in either the mitigation or exacerbation of neuroinflammatory responses.

In this line, recent studies have demonstrated that peripheral exosome administration can modify the expression of immune-response-related genes within the CNS, resulting in the induction of a proinflammatory phenotype in glial cells [85]. It has been proposed that this prolonged activation of microglia, mediated by enriched TLR ligands and cytokines in the enriched exosome fraction, may contribute to the loss of phagocytic function in senescent microglia, reducing their ability to clear beta-amyloid plaques and exacerbating neuronal damage [86]. In this line, altered exosomal cargo may compromise the functionality of the BBB, favoring peripheral immune cell infiltration and amplifying neurodegenerative inflammation [87]. This interplay underscores the notion that gut dysbiosis, in addition to inducing local changes in the microbiota, may directly influence Alzheimer’s progression by modulating exosomal activity and its impact on neuroinflammation [88].

Beyond their function in the immune response, the molecular composition of exosomes varies depending on the cellular origin of these vesicles and the surrounding inflammatory environment [89]. Exosomes derived from microglia in a state of pathological activation have a higher abundance of proteins related to dysfunctional autophagy (a cellular process where cells self-digest to survive starvation or other stresses) and inflammatory signaling [90]. It has been identified that these exosomes may contain markers of mitochondrial damage and ROS, which amplify neuroinflammation when internalized by neurons and astrocytes [91]. Conversely, astrocytic exosomes have been shown to contain molecules that exacerbate glutamatergic excitotoxicity and contribute to the synaptic loss that is characteristic of AD [92,93]. It has been suggested that these vesicles may lose their ability to transport essential trophic factors, such as brain-derived neurotrophic factor (BDNF), under conditions of chronic neuroinflammation, further contributing to synaptic dysfunction (Figure 2). These disparities in the molecular composition of these vesicles underscore their function as regulators of inflammation within the CNS, contingent on the nature of the inflammatory stimulus and the extent of intestinal dysbiosis present in the patient [42,94].

Conversely, recent studies have demonstrated the potential of modulating the gut microbiome to influence the regulation of exosomal communication and neuroinflammation. Proposed interventions, such as fecal microbiota transplantation (FMT) [95] or supplementation with SCFAs [96], have been suggested to potentially influence the composition of circulating exosomes, thereby reducing their inflammatory load and promoting a neuroprotective environment (Figure 2). However, further research is necessary to ascertain the clinical feasibility of this approach in AD, particularly in relation to the microbiota, exosomes, and neuronal inflammation.

However, it is imperative to meticulously deliberate on the presence of several translational barriers. The initial challenge in the effective delivery of exosome-based therapeutic agents to the CNS is the restricted permeability of the BBB. Unmodified vesicles exhibit low translocation rates in vivo, despite the implementation of surface engineering strategies [97]. Moreover, systemically administered exosome administration has been observed to elicit unintended immune responses, including complement activation or uptake by the mononuclear phagocyte system. This potential for immune stimulation may result in a reduction in bioavailability and may raise safety concerns [82,83]. Finally, the intrinsic heterogeneity of exosome preparations, arising from variable size distributions, donor-cell origins, and cargo compositions, introduces variability between batches, which compromises reproducibility and dosing consistency [98].

## 5. Diagnostic and Therapeutic Applications Based on Exosomes and Microbial Metabolites

Exosomes have emerged as a promising tool in the identification of biomarkers for early diagnosis of AD. Their presence in biological fluids such as blood, cerebrospinal fluid, and saliva allows for the analysis of their molecular content, providing key information about the pathophysiological state of the CNS [97]. In this line, CNS-derived exosome analysis has revealed the presence of specific metabolic signatures, including metabolites derived from the intestinal microbiota, which reflect the interaction between intestinal dysbiosis and disease progression [99,100]. Recent studies have demonstrated that specific microRNAs, proteins, and lipids transported by these vesicles can correlate with amyloid load and tau hyperphosphorylation, suggesting their potential as less invasive biomarkers of the disease. Furthermore, the detection of SCFAs in plasma-derived vesicles could provide a novel approach to evaluate the influence of the microbiome on the pathophysiology of AD [101,102].

Recent advancements in exosomal engineering have underscored the potential to enhance the therapeutic efficacy of SCFAs by encapsulating these metabolites within engineered exosome structures [103]. This strategy ensures targeted delivery to the CNS and protects SCFAs from metabolic degradation in circulation, thereby maximizing their neuroprotective and anti-inflammatory effects [104]. Moreover, the administration of exosome-based delivery systems, loaded with SCFA-rich molecular compositions, has been demonstrated to modulate glial activation and restore synaptic homeostasis in experimental models of AD [105,106]. Consequently, these findings underscore the potential of combining microbiota-targeted interventions with exosomal therapies to develop integrative approaches that address both systemic and CNS-specific pathological processes (Figure 2).

The manipulation of the intestinal microbiome has emerged as a promising strategy to modify exosomal composition and its associated impact on neuroinflammation [107]. The administration of probiotics and prebiotics has demonstrated significant benefits, including the restoration of microbial balance and the promotion of SCFA production [108]. These SCFAs possess anti-inflammatory and neuroprotective properties. Specifically, the administration of butyrate, a SCFA with epigenetic effects, has been observed to regulate the expression of genes involved in exosomal biogenesis and the modulation of microglia [109], thereby reducing the release of proinflammatory exosomes (Figure 2). Furthermore, it has been suggested that butyrate may influence the stability of the BBB, improving CNS homeostasis and reducing the infiltration of peripheral inflammatory signals [47,110]. The impact of butyrate on chromatin remodeling and the regulation of anti-inflammatory cytokine expression underscores its potential as a modulator of the gut–brain axis [111]. In addition, FMT has been investigated as a strategy to reconfigure the exosomal profile and restore neuroimmune homeostasis in experimental models of AD [112]. Observations have revealed that FMT can induce alterations in the molecular composition of exosomes, leading to a reduction in proinflammatory microRNAs and an enhancement in the transfer of trophic and antioxidant factors [113,114]. This suggests a potential indirect therapeutic approach to alleviate neuroinflammation induced by exosomes (Figure 2).

Exosomal engineering is an innovative approach that combines the manipulation of the microbiome with the modulation of the molecular cargo of the exosomes [115]. Strategies are being developed to design therapeutic exosomes with specific molecular profiles, capable of transporting SCFAs, neurotrophic factors, or inhibitors of amyloid aggregation to the CNS [116] (Figure 2). This approach seeks to take advantage of the natural ability of the exosomes to cross the BBB and act as vehicles for the selective delivery of bioactive compounds [117]. As mentioned, recent research has demonstrated that the administration of SCFA-enriched exosomes can reduce neuroinflammation and enhance synaptic function in neurodegeneration models [84,86]. However, further validation is required for its application in humans. Furthermore, the development of genetically modified exosome-based therapies that can inhibit inflammatory protein expression or enhance neuroprotective molecule release holds significant potential for personalized Alzheimer’s therapy [118]. Several exosomal engineering strategies have been proposed, including the modification of the membrane to improve its cellular tropism, the insertion of transport proteins to enhance the delivery of specific metabolites, and the optimization of its stability in circulation to prolong its half-life in the organism [119,120].

The integration of these approaches provides a novel perspective for the development of targeted therapies in AD. The combination of strategies that modulate the intestinal microbiome with exosome-based interventions could allow the design of personalized treatments, adapted to the metabolic and exosomal profile of each patient. However, the standardization of exosomal isolation and characterization methods, as well as the validation of specific biomarkers, are pending challenges that must be addressed in future studies to facilitate the clinical translation of these emerging strategies. Furthermore, the identification of possible interactions between engineered exosomes and other components of the CNS will be essential to understand their long-term safety and efficacy, opening new opportunities for the application of combination therapies that include immunomodulation, neuroprotection, and the restoration of the intestinal microbiota as a comprehensive therapeutic approach.

## 6. Challenges and Future Perspectives

Despite the increasing interest in exosome research and their relationship with the gut microbiota in AD, significant technical and methodological challenges must be addressed before their clinical implementation. A primary challenge is the standardization of protocols for isolating and characterizing exosome samples [121]. Current techniques, including ultracentrifugation, immunoprecipitation, and microfluid-based approaches, have limitations in terms of purity, specificity, and reproducibility [98,122]. The absence of standardization in the field presents significant challenges, including the difficulty involved in comparing results across studies and the validation of specific exosomal biomarkers for the early detection of AD. Furthermore, the characterization of exosomal metabolites derived from the microbiome remains an area of active research, necessitating advanced methodologies that enable precise identification of bioactive compounds transported by these vesicles and their influence on the pathophysiology of the disease [123].

Another significant challenge pertains to the interpretation of the influence of the gut microbiome on exosomal modulation. Although dysbiosis has been demonstrated to alter the biogenesis and content of exosomes, the functional heterogeneity of these vesicles in different cell types complicates the identification of universal patterns [124]. Microglia-derived exosomes may exert a distinct impact on neuroinflammation compared to neuronal or astrocytic exosomes, suggesting that their role in Alzheimer’s pathogenesis is highly dependent on the physiological context [125]. Furthermore, the impact of individual factors, such as diet, antibiotic use, or genetic predisposition, adds additional sources of variability, making it difficult to extrapolate preclinical findings to clinical practice.

Different studies have highlighted the neuroprotective potential of exosomes and SCFAs, yet several recent meta-analyses have documented limited or heterogeneous effects of interventions on the gut microbiota in patients with AD or MCI. For instance, a systematic review and meta-analysis identified no consistent changes in cognitive function or microbial composition following probiotic or mixed treatments in AD/MCI cohorts [126]. In the domain of exosomal biomarkers, Soares Martins et al. (2018) [127] conducted a comparative analysis of precipitation-based isolation methods and column-based methods. This study revealed discrepancies in the proteomic profiles detected by mass spectrometry.

Future research should focus on advancing emerging technologies that enable more-precise characterization and targeted manipulation of exosome-related mechanisms in the context of AD. Nanotechnology and artificial intelligence (AI) are rapidly emerging as cutting-edge tools in this domain, enabling more-precise analysis of exosomal molecular profiles and facilitating the identification of biomarkers with enhanced specificity and sensitivity [128]. Machine learning algorithms, for instance, have the potential to enhance the interpretation of omics data, thereby unveiling intricate correlations between microbiota composition, exosomal cargo, and neuroinflammatory progression. These technologies are poised to address current methodological limitations, paving the way for more-efficient integration of exosome-based diagnostics in clinical practice [129].

The bioengineering of exosomes represents a promising avenue for personalized therapeutic interventions. Recent advancements include the development of exosome delivery systems capable of traversing the BBB and selectively targeting CNS cells. These engineered vesicles can be optimized to carry anti-inflammatory agents, neurotrophic factors, or metabolic modulators derived from SCFAs, which may restore neuroimmune balance and mitigate neurodegeneration [130]. Furthermore, combining microbiome modulation with exosomal therapies offers a holistic approach to addressing the multifactorial nature of AD. By simultaneously targeting systemic and CNS-specific mechanisms, this strategy has the potential to yield more robust and sustainable therapeutic outcomes.

A comprehensive approach for future therapeutic strategies could include the combination of gut microbiota modulators with exosomal therapies designed to interfere with Alzheimer’s pathological processes. The integration of these approaches will enable the assessment of their impact on disease progression and support the development of validation protocols for clinical trials. Furthermore, research into the interaction between exosomes and other cellular systems, such as peripheral immune cells and the BBB, will provide a more complete view of their role in pathogenesis and open new therapeutic opportunities. As methodological and technological challenges are addressed, the potential of exosome-based diagnostics and therapeutic interventions in AD can be realized through the development of innovative and personalized clinical strategies.

## 7. Materials and Methods

The present narrative review is an exhaustive analysis of the extant evidence regarding the involvement of exosomes in the pathogenesis, diagnosis, and therapeutic potential of Alzheimer’s disease, with a particular focus on their modulation by the intestinal microbiota and its bacterial metabolites in neuroinflammation. In this updated approach, studies that explicitly address not only the role of these nanovesicles in AD but also the impact of gut dysbiosis, the gut–brain axis, and metabolites such as SCFAs on exosome biogenesis and function were considered. This included original research, comprehensive reviews, and clinical and preclinical studies that offered significant data on the function, characteristics, and applications of these nanovesicles in the disease. To ensure a comprehensive overview, English-language publications from 2015 to 2025 were included, encompassing both recent studies and fundamental research that has laid the foundations in the field. The search was restricted to English-language publications because leading indexing platforms (e.g., PubMed, Web of Science) predominantly cover journals in English. This ensured consistent critical appraisal by the authors. Articles that did not address the interplay between exosomes and the intestinal microbiota, or that focused on extracellular vesicles in contexts other than neurodegenerative diseases, were excluded.

The following scientific databases were utilized in the literature search: ScienceDirect, Medline, Wiley, Scopus, Web of Science, and PubMed. The search strategy was formulated through the implementation of Boolean operators and combinations of key terms, with the objective of ensuring comprehensive coverage of the subject matter. Initially, primary terms such as “exosome”, “exosomes”, and “extracellular vesicles” were used. Expressions related to Alzheimer’s disease were combined with the terms “Alzheimer”, “Alzheimer’s disease”, and “dementia”. Furthermore, to capture studies addressing the influence of the gut microbiota, additional terms such as “gut microbiota”, “intestinal microbiota”, “microbiome”, “gut–brain axis”, “dysbiosis”, and “SCFAs” were incorporated. An example search string was: (“exosome” OR “exosomes” OR “extracellular vesicles”) AND (“Alzheimer” OR “Alzheimer’s disease” OR “dementia”) AND (“pathogenesis” OR “biomarker” OR “biomarkers” OR “diagnosis” OR “therapy” OR “neuroinflammation” OR “neurodegeneration”) AND (“gut microbiota” OR “intestinal microbiota” OR “microbiome” OR “gut–brain axis” OR “dysbiosis” OR “SCFAs”). This strategy was adapted according to the syntax requirements of each database.

The selection process for literature was conducted in a series of stages. Initially, a preliminary search was executed using a previously described strategy, which yielded a substantial set of potentially relevant articles. Subsequently, the titles and abstracts of these articles were evaluated to eliminate those that did not meet the established inclusion criteria. The studies that were pre-selected were then read in their entirety to confirm their relevance and methodological quality, facilitating the extraction of key data related to the objectives of this review. The extracted information included the objectives, the methodology used, the relevant findings, and the limitations of each study, allowing the information to be organized into thematic categories that contributed to the construction of a coherent and critical narrative about the role of exosomes in AD. Finally, a cross-validation of the information collected was carried out to guarantee the consistency and integrity of the data presented in this work.

By integrating search terms and inclusion criteria that emphasize the role of the microbiota and its metabolites, this review aims to provide an updated and rigorous vision of the advances, challenges, and future perspectives in the study of exosomes in AD. This approach contributes to a comprehensive understanding of the intricate interplay between peripheral metabolic signals and central neurodegenerative processes, as well as the novel therapeutic avenues that may arise from this integration.

## 8. Conclusions

Alzheimer’s disease remains a significant challenge in the field of neuroscience due to the intricacy of its pathogenic mechanisms and the paucity of effective disease-modifying therapies. The hallmarks of this debilitating condition are the buildup of amyloid-β and the hyperphosphorylation of tau, accompanied by a persistent neuroinflammatory response. Therapeutic interventions aimed at these processes have yielded inconsistent results, largely due to the complexity of intervening in early stages and the heterogeneity of the factors involved in the progression of the disease.

Extracellular vesicles (i.e., exosomes), as pivotal mediators in cellular communication within the CNS, have been implicated in the propagation of pathological proteins, as well as in the regulation of neuronal homeostasis and glial response. Their dual role, encompassing both the dissemination of neurodegenerative processes and the promotion of neuroprotective mechanisms, has led to a surge of scientific interest in these vesicles. The recent evidence indicates that these vesicles function as vectors in the progression of AD by facilitating the transportation of pathological molecular cargo, including amyloid-β oligomers and hyperphosphorylated tau aggregates, between neural cells.

Recent findings have further expanded this perspective by demonstrating that the gut microbiota plays a critical role in modulating exosome biogenesis and cargo. Dysbiosis and disruptions in the gut–brain axis contribute to a pro-inflammatory environment that alters the molecular composition of exosomes, thereby influencing neurodegenerative processes. This interplay suggests that interventions aimed at restoring a healthy microbiome—through probiotics, prebiotics, or FMT—and enhancing the production of neuroprotective metabolites such as SCFAs may complement exosome-based strategies.

The ambivalent nature of this character has given rise to a line of research that explores the diagnostic and therapeutic potential of exosome analysis. In the domain of diagnostics, the isolation of exosomes in biofluids such as plasma or cerebrospinal fluid could facilitate the early detection of specific biomarkers associated with the preclinical stages of the disease, thereby anticipating cognitive deterioration before clinical manifestation.

Regarding their therapeutic potential, exosome therapy offers a cutting-edge system for the directed delivery of drugs and biomolecules to the brain, thereby partially surmounting the restrictions imposed by the BBB. Moreover, the engineering of exosomes enables the modulation of their molecular charge to interfere with the spread of pathological proteins and to regulate neuroinflammation. Initial preclinical findings are encouraging, though further validation is required regarding their clinical application, including the need for larger-scale trials to assess safety and efficacy.

Recent data indicate that alterations in microbial composition and the resulting pro-inflammatory environment can modulate the biogenesis and cargo of exosomes, thereby influencing their role in neurodegenerative processes. This finding suggests a potential for complementary therapeutic strategies that combine interventions aimed at restoring a healthy gut microbiome with exosome-based approaches. This strategy offers a more integrated and potentially effective means to attenuate disease progression.

Despite the nascent stage of research in the field of exosomes in AD, their potential as biomarkers and their role as personalized therapeutic platforms position them as a promising area in the search for new diagnostic and therapeutic solutions for this debilitating disease. However, overcoming current technical limitations and enhancing our understanding of exosomal biology are essential for their effective clinical implementation. Nonetheless, the lack of standardized isolation and analysis methods remains a fundamental obstacle to their translation into practice.

## Data Availability

No new data were created or analyzed in this study.
